# Supplementary figures and images for: E(3) equivariant graph neural networks for robust and accurate protein-protein interaction site prediction
Source: PLoS Comput Biol. 2023 Aug 31;19(8):e1011435. doi: 10.1371/journal.pcbi.1011435 (PMC10499216; doi:10.1371/journal.pcbi.1011435)

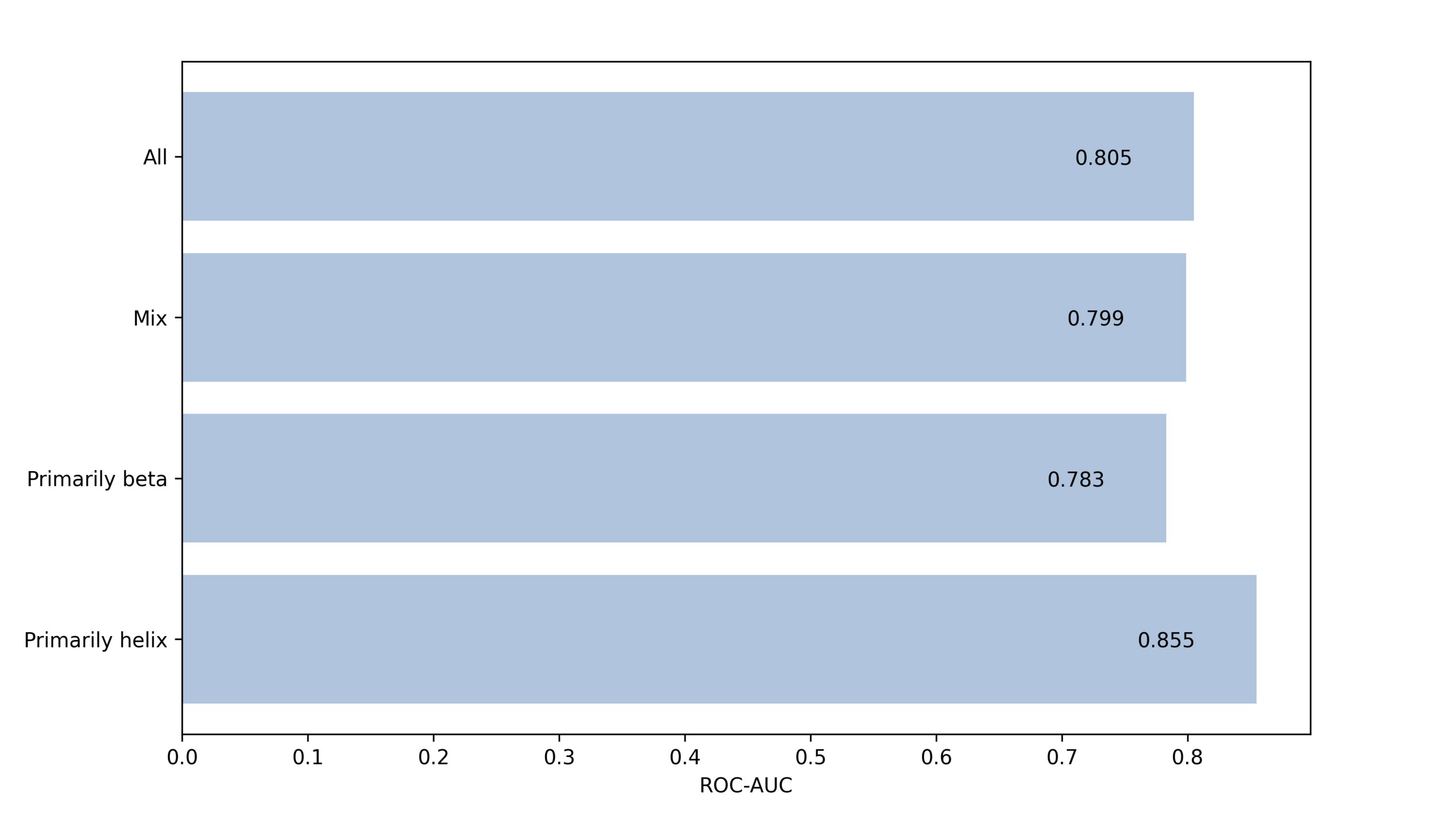

Supplement: S1 Fig — ROC-AUC scores achieved by EquiPPIS grouped by secondary structure content (’Primarily helix’, ’Primarily beta’, and ’Mix’) as well as the overall ROC-AUC (’All’) in the Test_60 set. (TIF) [file pcbi.1011435.s001.tif]

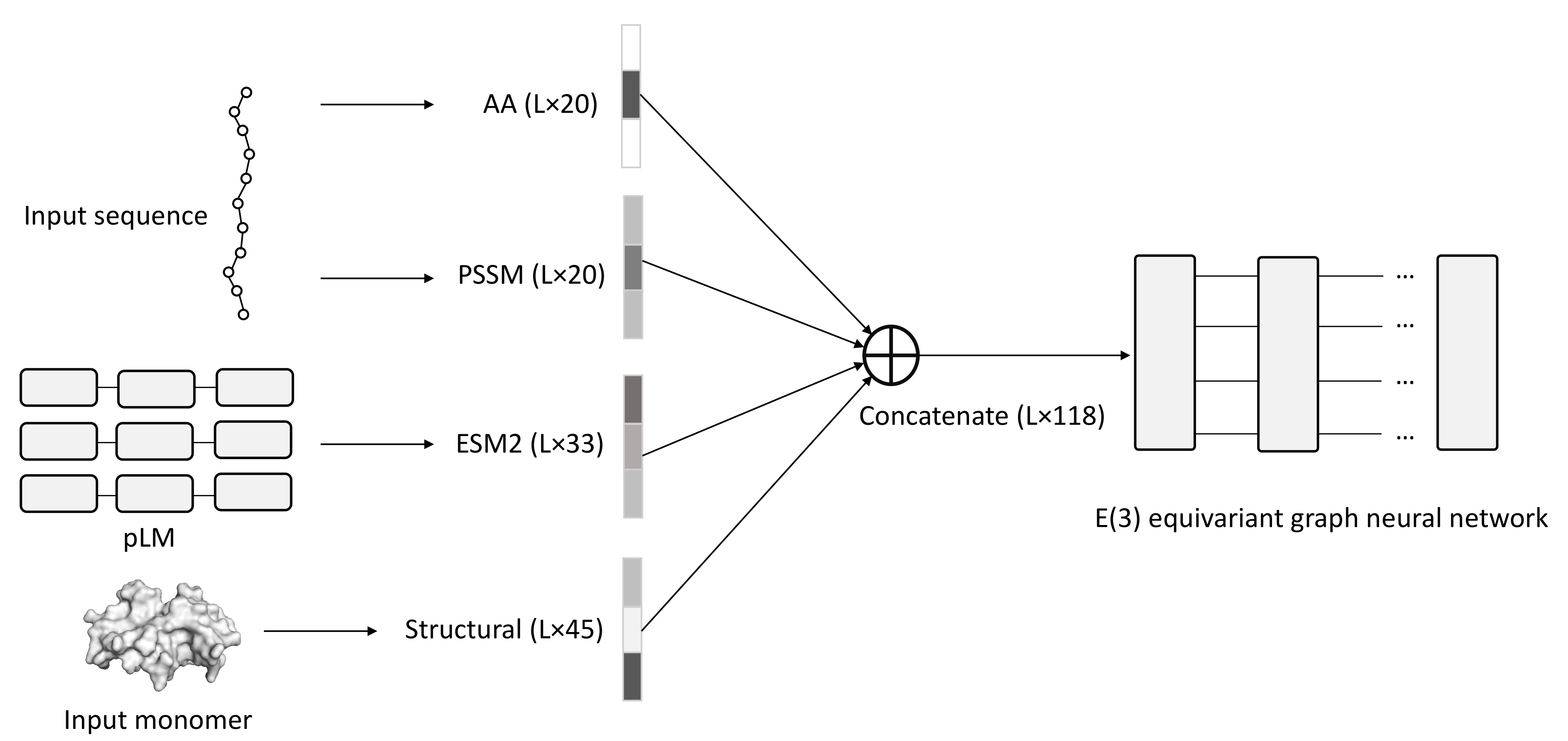

Supplement: S2 Fig — The sequence-based amino acid (L×20 feature set), PSSM (L×20 feature set), and ESM2 (L×33 feature set) features are concatenated with the structure-based node features (L×45 feature set), leading to a total of L×118 features, which serves as an input to the E(3) equivariant graph neural networks. (TIF) [file pcbi.1011435.s002.tif]
